# Non-prescription antibiotics dispensing for common infections using simulated patients

**DOI:** 10.1016/j.rcsop.2026.100791

**Published:** 2026-04-24

**Authors:** Arsha Khan, Muhammad Awais Fareed, Daniyal Rauf, Muhammad Ameer Usama, Mubashra Rehman, Neha Sheerin, Atif Usman, Zainub Khan, Imran Masood, Jamshaid Akbar, Ali Ahmed

**Affiliations:** a Department of Pharmacy, Faculty of Pharmacy, The Islamia University of Bahawalpur, Pakistan; b Department of Pharmacy Practice, Faculty of Pharmacy, The Islamia University of Bahawalpur, Pakistan; c Division of Infectious Diseases and Global Public Health, School of Medicine, University of California San Diego (UCSD), La Jolla, CA, USA; d Perelman School of Medicine, University of Pennsylvania, Philadelphia, Pennsylvania, USA

**Keywords:** Non-prescription dispensing, Common infections, Simulated patient, Antimicrobial resistance

## Abstract

**Background:**

Non-prescription antibiotic dispensing at community drugstores is a widespread public health concern, particularly in low and middle-income countries. This practice promotes irrational antibiotic use that can hasten the emergence and spread of antibiotic resistance.

**Objectives:**

This simulated patient study aimed to assess the rate and dispensing patterns of non-prescription antibiotics for common infections, and concurrently document the types and classes of antibiotics dispensed.

**Methods:**

A cross-sectional study using the simulated patient method was conducted in Bahawalpur, Pakistan, for a period of one month starting from May 28 to June 29, 2025, and was reported in accordance with the CRiSPHe Statement (checklist for reporting research using a simulated patient methodology in Health). Four common simulated scenarios, flu, diarrhea, sore throat, and urinary tract infections (UTIs) were presented. Descriptive statistics were used to determine frequencies and percentages, while the association between non-prescription antibiotic dispensing and independent variables was determined by using inferential statistics. *P*-values<0.05 were considered significant.

**Results:**

Of the total 175 drugstores visited, 96.5% of them dispensed antibiotics without a prescription. The most commonly dispensed antibiotics were ciprofloxacin (31.7%), levofloxacin (15.6%), and metronidazole (10.8%). Most of the antibiotics (79%) were dispensed at the first level of demand, with 89.5% dispensed single antibiotic, while 10.2% dispensed two antibiotics in a single visit. Qualified personnel were present at only 9 (5.2%) drugstores. Drugstores located in front of the hospitals were 10.1 times less likely to dispense antibiotics without a prescription than those located in the community settings (95%CI:0.016–0.619, AOR:0.099).

**Conclusion:**

Non-prescription antibiotic dispensing is a common practice, fueling antibiotic resistance. Strengthening regulations, enforcing prescription-only policies, and increasing public awareness are essential to ensure safe and rational antibiotic use.

## Highlights

• About 96.5% of drugstores dispensed antibiotics without a prescription, primarily at the first level of demand.
• Ciprofloxacin (Watch group) was most dispensed, risking dependency on Reserve drugs.
• Absence of qualified staff (Pharmacist) resulted in inadequate clinical assessment, patient counseling and medical referrals.
• Targeted staff training, frequent inspections, and policy reviews are needed to mitigate AMR risks.

## 1 Introduction

Antibiotics, initially recognized as the magic bullets, discovered in the 20th century, were one of the biggest breakthroughs in medical research against bacterial infections.^1^ As a pillar of modern medicine, antibiotics have played a crucial role in lowering mortality and remain essential for both invasive surgery and intricate treatments such as chemotherapy.^2^ But in the 21st century, due to the pervasive proliferation of superbugs, antimicrobial resistance (AMR) has emerged as a critical global health concern, rendering antibiotics less effective against many infections.^3^ It is estimated that about 700,000 people die annually due to infections caused by drug-resistant strains, and if it remains unaddressed, this annual fatality rate may escalate to 10 million by 2050.^4^ In addition to its devastating impact on health, the World Bank projected that AMR would lead to a 3.8% decline in world gross domestic product (GDP) by 2050, pushing an additional 24 million people below the poverty line.^5^ This crisis is disproportionality fueled by the irrational use of antibiotics, of which the dispensing of these drugs without a prescription is one of the chief drivers in community settings.^6^

The practice of non-prescription antibiotic dispensing and the resulting misuse are highly centered on community drugstores, which often serve as the initial, and sometimes the only point of contact for health concerns.^7^ The World Health Organization (WHO) highlights that about 93% of antibiotic access comes from community drugstores,^8^ with more than 50% of all antibiotics sold without a prescription.^9^ This unregulated access to antibiotics, particularly in underprivileged areas, paves the way for rampant public practice of self-medication leading to drug toxicity, AMR, increased hospital stay, and treatment failure.^10^ Pharmacists are perfectly positioned as frontline healthcare providers with the mandate to limit inappropriate use and to foster safe and rational use of these medications.^11^ However, this role of pharmacist is compromised by two issues: first, the absence of pharmacists in a significant number of operating pharmacies,^12^ and second is the professional breach where the qualified, designated as the gatekeepers against antibiotics misuse, are themselves fostering the problem. Studies indicate that the inappropriate utilization and practices of self-medication with antibiotics are, in the majority of cases, connected to the pharmacists' frequent and unsuitable antibiotic dispensing practices.^13^, ^14^ This global problem is more critical in low and middle-income countries (LMICs),^15^ with 1.5 times higher rates as compared to developed countries, probably due to the lack of a national action plan and poor implementation of existing regulations that make it even more dreadful.^16^

Among LMICs, Pakistan stands as the third top nation for antibiotic use, with the consumption rate having surged by 65% from 2000 to 2015.^17^ It is estimated that in Pakistan, 35,000 people use antibiotics daily.^18^ More than half of the patients in rural areas use antibiotics without a prescription,^19^ and above 35% of antibiotics are dispensed by urban drugstores without a prescription, highlighting a severe lack of regulations.^20^ The Drugs Act 1976 of Pakistan states that antibiotics are not over-the-counter (OTC) medicines and prohibits their dispensing without a prescription.^21^ Additionally, recognizing antibiotics misuse as a threat to public health and the One Health framework, Pakistan introduced the Antimicrobial National Action Plan to ensure a coordinated and sustainable response by integrating efforts across various sectors.^22^ For this, a steering committee was established, empowered to identify key stakeholders and experts for decision-making, assess the current status of AMR, develop policies, and formulate recommendations.^23^ But evidence shows that the enforcement of these regulations is still violated, as studies conducted in different areas of the country revealed that most of the drugstores provide antibiotics without a prescription.^24^, ^25^, ^26^

There is a scarcity of evidence on the pattern of antibiotics dispensing without prescription using a simulated patient (SP) technique, which is internationally recognized for outcome measures in pharmacy practice research, and it has been used to assess the non-prescription sale of antibiotics at community pharmacies worldwide.^27^ This technique is advantageous as it diminishes the Hawthorne effect.^28^ Only a few SP studies have been conducted in Pakistan,^24^, ^29^ but southern Punjab region is still unaddressed. This region has low literacy rate as compared to other areas,^30^ and evidence demonstrates that there is a high rate of non-prescription antibiotic sales in the communities with low literacy rates.^15^, ^31^ To address this gap, this study employs the standardized SP technique.^32^ The core purpose of this study is two-fold: first, to measure the rate with which antibiotics are dispensed without a prescription for four common infections, i.e., flu, acute diarrhea, sore throat, and urinary tract infections (UTIs) in adults; and second, to catalogue the pattern of non-prescription antibiotic dispensing for these infections. The findings will provide the necessary evidence base for designing targeted, effective interventions to ensure policy compliance and enhance global health security.

## 2 Methodology

### 2.1 Study design and study settings

A cross-sectional study was conducted in Bahawalpur, a major urban center in Southern Punjab, Pakistan. This city is known for its diverse population and as an essential hub for regional healthcare services.^33^ The study employed a simulated patient (SP) methodology, and was reported in accordance with the CRiSPHe Statement (checklist for reporting research using a simulated patient methodology in Health).^32^ Data were obtained from drugstores holding a valid license issued by the Punjab Pharmacy Council (PPC) and the Central Drug and Store Licensing Board, under the Health and Population Department.

### 2.2 Sampling and sample size determination

**Fig. 1:**
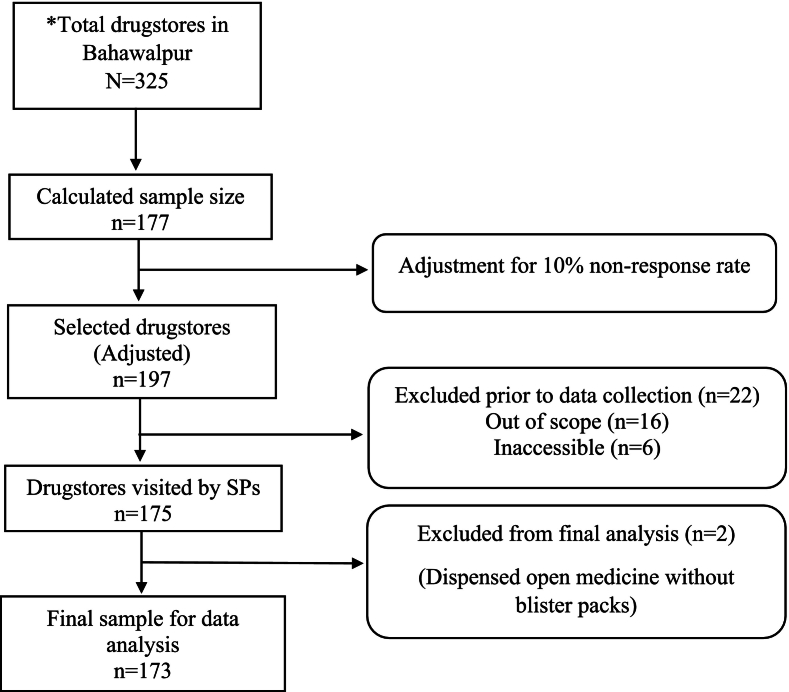
Flow diagram of drugstore sampling and selection process. * Total drugstores in Bahawalpur when assessed online.

The sampling procedure is detailed visually in Fig. 1. A list of drugstore names was assessed online on May 18, 2025,^34^ and a total of 325 drugstores were found to be situated in Bahawalpur. A sample size of *n* = 177 was calculated using the Raosoft sample size calculator, incorporating a 95% confidence interval, 5% margin of error, and 50% response distribution.^35^ However, considering a 10% non-response rate, the final sample size was adjusted to *n* = 197 drugstores. A systematic random sampling method was employed to identify the drugstores, utilizing a fixed interval of two. Before data collection, 22 drugstores were excluded as they did not meet the scope of the study (*n* = 9 veterinary pharmacies, and *n* = 7 surgical suppliers) and were inaccessible (*n* = 6 closed at the time of the visit). Therefore, the simulated visits were ultimately conducted at 175 drugstores. During data analysis, a further two drugstores were excluded as they provided vague data. Hence, the final analytical sample comprised 173 drugstores.

### 2.3 Simulated scenarios

This study presents four adult simulated clinical scenarios, i.e., flu, acute diarrhea, sore throat, and urinary tract infections (UTIs), selected for their high prevalence and association with antibiotic overuse at the community drugstores.^11^ The rationale for simulated scenarios and expected outcomes is provided in Table 1. All other relevant details regarding the simulated scenarios are provided in Supplementary File 1.

**Table 1:** Simulated scenarios.

Cases	Symptoms	Additional information (if required)	Rationale of the selected scenario	Expected results
1	A 25-year-old pseudo-patient is experiencing symptoms including coughing, weakness, throat irritation, congestion, headaches, sweating, chills, general discomfort, a runny nose, trouble breathing, difficulty sleeping, and myalgia, indicating the need for an appropriate antibiotic treatment.	1. No known allergies in the past. 2. Not taking any medications at the moment. 3. No existing health conditions. 4. No physician visits. 5. No signs of fever. 6. No relatives are experiencing similar symptoms at this time.	Influenza, categorized as a URTI, is a common self-limiting viral infection for which antibiotics are often misused.^36^	Antibiotics should not be dispensed. Patients are advised to rest and may use over-the-counter medications, such as acetaminophen, for flu-like symptoms. If symptoms persist for a week or worsen, consulting a physician is recommended.
2	A 25-year-old pseudo-patient (male or female) is experiencing mild symptoms, including nausea, vomiting, abdominal cramping, and increased stool volume, characterized by non-bloody, watery stools, prompting them to visit the toilet every 3 to 4 h; they are seeking medication to alleviate these symptoms.	1. Do not have any prior allergies. 2. No current medicine. 3.No co-morbidities. 4. The stool didn't contain any mucus or blood. 5. Patient feels, lack of appetite. 6. Not visited any physician. 7. There is no sign of fever. 8. Currently, no family member is having similar symptoms.	Over 60% of pharmacies in Pakistan dispense antibiotics without prescriptions for diarrheal diseases.^29^	No antibiotics should be dispensed. Patients are advised to take Oral Rehydration Solution (ORS) and should receive clear instructions on its preparation. Emphasis should be placed on proper hygiene, including handwashing. Patients should also be encouraged to see a physician if diarrhea lasts more than a week or worsens.
3	A 25-year-old pseudo-patient (male/female) presents with dryness and a burning sensation in the throat, difficulty swallowing, fever, and swollen mucosa, having experienced these symptoms for the past three days, and is requesting medication to relieve his/her discomfort.	1. No known allergies in the past. 2. Not taking any medications at the moment. 3. No existing health conditions 4. The mouth is rinsed with salt water, but it didn't make much difference 5. The patient has not taken any medication. 6. There is no cough present. 7. No signs of headache. 8. Not visited any physician.	Although selling antibiotics without a prescription is prohibited in Pakistan, they were dispensed in 69.1% of cases for upper respiratory tract infections (URTIs).^14^	Antibiotic dispensing is not recommended. The SP should gargle with saline and may use an over-the-counter antipyretic, such as paracetamol, for fever. They should also be advised to seek medical attention if symptoms persist beyond one week or worsen.
4	The pseudo-patient reports fever, itching, burning in the genital area, blister formation, pain during urination, flank pain, and chills, which started two days ago. She has been drinking more water than usual and requests medication to alleviate her symptoms.	1.No known allergies in the past. 2. Not taking any medications at the moment. 3. No existing health conditions. 4. Not tried anything. 5. Low-grade fever. 6. No back pain. 7. She is not pregnant/not expecting to be pregnant shortly. 8. Not visited any physician. 9. Last time had the same problem about 12 months ago	In about 88.1% of simulated client scenarios, women in Pakistan received antibiotics without a prescription for UTIs.^24^	Antibiotics should not be dispensed. The SP should be recommended to see a physician.

### 2.4 Data collection team

The visits were conducted by four trained SPs, of whom two were males and two were females. They were all Pharm-D final year students enrolled in a public sector university. To ensure high-quality and consistency across the study, each SP presented only one assigned scenario throughout the study to minimize inter-scenario variability. Before data collection, all SPs underwent a training session directed by a senior clinical researcher. As every SP was assigned to present a specific clinical scenario, training was conducted individually, with each SP attending a separate one-day training session dedicated to their assigned case. These training sessions were conducted in Urdu, which is a primary language of communication in community settings of Pakistan. A mixed approach (role-plays, discussion, and peer reflection) was utilized during training to ensure proficiency in scenario delivery, data collection, and to complete the study forms. Role-play activities enabled SPs to practice realistic symptom presentation and to rehearse typical counter questions anticipated by drugstore staff. Discussions were made on potential challenges during visits, such as maintaining a natural demeanor, responding to unexpected questions, and leaving the drugstore without buying the medication. Peer reflection activities allowed SPs to present and get constructive feedback, which helped them to refine scenario delivery and ensure proper completion of a structured data collection form. Following the training session, the lead researcher observed a final role-play of each SP. SPs were considered field-ready when they were able to present their designated scenario consistently and respond appropriately to common counter questions without prompting. A structured data collection form was adapted and utilized to gather information during the visits. Both the visiting process and data collection tool were tested and improved through a pilot survey using 10% of the total sample size; however, the findings from the pilot phase were not included in the final analysis.

### 2.5 Data collection procedure

**Fig. 2:**
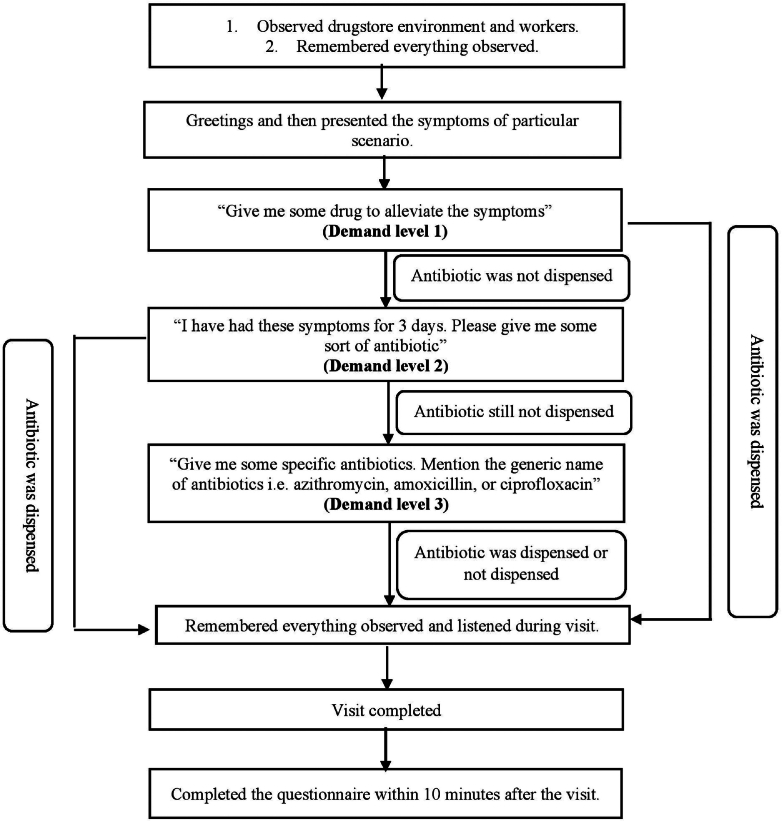
Simulated patient visiting process flowsheet.

For convenience, drugstores were grouped geographically, allowing the SPs to visit multiple drugstores in the same area on the same day. Each drugstore receives a single visit by a single SP. The SPs first visited the drugstore, observed some basic details about the store and its staff, and then described their medical conditions. The SPs used three levels of direct product request (DPR) until the antibiotics were either dispensed or refused. Demand level 1 (DL-1): The SPs explained symptoms to the drugstore staff and asked for suitable medication. Level 2 (DL-2): If the antibiotic was not dispensed, the SPs requested, “I have had these symptoms for three days, could you please give me antibiotics?” Level 3 (DL-3): “Give me antibiotic i.e,. azithromycin, amoxicillin, or ciprofloxacin.” The SPs remembered everything and made an excuse for not buying the antibiotic before leaving the pharmacy. To improve the accuracy of the collected data, the SPs were encouraged to fill out the form within 10 min of leaving the drugstore. The visiting process of SPs to drugstores is demonstrated in Fig. 2.

### 2.6 Statistical analysis

Data were analyzed using Statistical Package for Social Sciences (SPSS) version 25. Descriptive statistics were used to determine frequencies and percentages. The Pearson's chi-square test (Χ^2^) and bivariate logistic regression were employed to determine the relationship between various dependent and independent variables. A significance level of *p* < 0.05 was established. In instances where one or more cells had an expected count of less than five, Fisher's exact test was applied. The variables that were found to be statistically significant in bivariate analysis were subsequently included in a multivariate logistic regression model.

### 2.7 Ethical approval

Ethical approval was obtained from the Pharmacy Human Ethics Committee (PHEC), the Islamia University of Bahawalpur, Pakistan (Ref No. 244–2025-/PHEC, May 12, 2025). The names of drugstores and identification numbers were kept confidential. All individual-level findings were maintained as confidential, and only aggregate research data were shared.

## 3 Results

### 3.1 Characteristics of drugstores and their staff

A total of 175 drugstores were visited. However, the data from 173 visits were included in the study as two stores provided ambiguous data; dispensed open medicines without blister packs, which prevented identification of the antibiotics. Among these, 93 (53.8%) were pharmacies, and 80 (46.2%) were medical stores. The number of independently working drugstores, and the chain pharmacies surveyed were 94.2% (*n* = 163) and 5.8% (*n* = 10), respectively. Most of the drugstores (*n* = 155; 89.6%) were located in community settings. Qualified personnel were present at only 5.2% (*n* = 9) of drugstores. All of the visited drugstores were staffed exclusively by males (100%), with the majority of them (*n* = 103; 59.5%) being over 30 years (Table 2).

**Table 2:** Characteristics of drugstores and their staff.

Demographics	N (%)
Drugstore category	
Pharmacy	93 (53.8%)
Medical store	80 (46.2%)
Type of drugstore	
Individual	163 (94.2%)
Chain	10 (5.8%)
Location of pharmacy	
Community setting	153 (88.4%)
In front of the hospital	20 (11.6%)
Gender of staff	
Male	173 (100%)
Female	0
Age of staff	
Below 30 years	70 (40.5%)
Above 30 years	103 (59.5%)
Qualified person on duty	
Yes	9 (5.2%)
No	164 (94.8%)
Antibiotic dispensed without a prescription	
Yes	167 (96.5%)
No	6 (3.5%)
Demand level at which antibiotics are dispensed	
Demand level 1 (DL-1)	132 (79%)
Demand level 2 (DL-2)	20 (12%)
Demand level 3 (DL-3)	15 (9%)
Number of antibiotics dispensed	
One	150 (89.8%)
Two	17 (10.2%)

### 3.2 Dispensing of non-prescription antibiotics

**Fig. 3:**
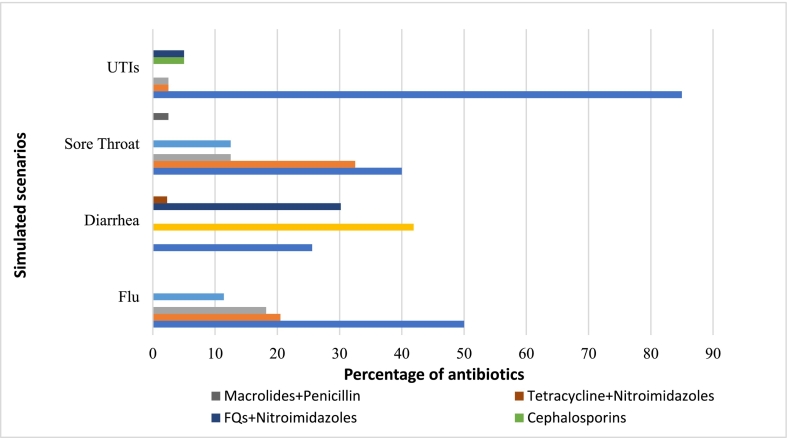
Class of antibiotics dispensed without a prescription.

A majority (96.5%) of the drugstores dispensed antibiotics without a prescription. Among these, 88 were pharmacies and 79 were medical stores, with no significant difference observed in their dispensing practices (*p* = 0.219). During a single visit, 89.8% (*n* = 150) drugstores dispensed one antibiotic, while 10.2% (*n* = 17) drugstores dispensed two antibiotics. The antibiotics most frequently sold by the drugstores were ciprofloxacin (31.7%), levofloxacin (15.6%), and metronidazole (10.8%). About 79% (*n* = 132) of drugstores offered OTC antibiotics at the first level of demand, 12% (*n* = 20) at the second level of demand, and 9% (*n* = 15) at the third level of demand. At demand level 3, ciprofloxacin (*n* = 7) was the most frequently dispensed antibiotic, followed by amoxicillin (*n* = 5) and metronidazole (n = 2). Of the six drugstores that did not dispense antibiotics, one stated that the issue did not require antibiotics, and five referred patients to a physician. The most commonly dispensed antibiotics for each simulated scenario are given in Table 3. The class of antibiotics most commonly dispensed without a prescription for each simulated scenario is given in Fig. 3.

**Table 3:** Antibiotics dispensed without prescription.

	Total (*n* = 173)	Flu (*n* = 45)	Diarrhea (*n* = 44)	Sore Throat (*n* = 40)	UTIs (n = 44)
Antibiotic dispensed	167 (96.5%)	44 (97.8%)	43 (97.7%)	40 (100%)	40 (90.9%)
Antibiotic not dispensed	6 (3.5%)	1 (2.2%)	1 (2.3%)	N/A	4 (9.1%)
Demand level 1 (DL-1)	132 (79%)	41 (93.2%)	35 (81.4%)	37 (92.5%)	19 (47.5%)
Demand level 2 (DL-2)	20 (12%)	1 (2.3%)	7 (16.3%)	N/A	12 (30%)
Demand level 3 (DL-3)	15 (9%)	2 (4.5%)	1 (2.3%)	3 (7.5%)	9 (22.5%)
Ciprofloxacin	53 (31.7%)	9 (20%)	11 (25%)	2 (5%)	31 (70.5%)
Levofloxacin	26 (15.6%)	11 (24.4%)	N/A	13 (32.5%)	2 (4.5%)
Metronidazole	18 (10.8%)	N/A	18 (40.9%)	N/A	N/A
Azithromycin	14 (8.4%)	8 (17.8%)	N/A	5 (12.5%)	1 (2.3%)
Ciprofloxacin + Metronidazole	14 (8.4%)	N/A	12 (27.3%)	N/A	2 (4.5%)
Amoxicillin	10 (6%)	5 (11.1%)	N/A	5 (12.5%)	N/A
Doxycycline	9 (5.4%)	5 (11.1%)	N/A	4 (10%)	N/A
Erythromycin	9 (5.4%)	1 (2.2%)	N/A	8 (20%)	N/A
Co-Amoxiclav	4 (2.4%)	3 (6.7%)	N/A	N/A	1 (2.3%)
Ofloxacin	2 (1.2%)	2 (4.4%)	N/A	N/A	
Moxifloxacin	2 (1.2%)	N/A	N/A	1 (2.5%)	1 (2.3%)
Cefixime	2 (1.2%)	N/A	N/A	N/A	2 (4.5%)
Erythromycin + Co-amoxiclav	1 (0.6%)	N/A	N/A	1 (2.5%)	N/A
Oxytetracycline	1 (0.6%)	N/A	N/A	1 (2.5%)	N/A
Levofloxacin + Metronidazole	1 (0.6%)	N/A	1 (2.3%)	N/A	N/A
Doxycycline + Metronidazole	1 (0.6%)	N/A	1 (2.3%)	N/A	N/A

### 3.3 Dispensing of non-prescription antibiotics for Flu

SPs presented a flu case at 45 drugstores, and antibiotics were dispensed by 44 of them. The levels of demand for non-prescription antibiotics were as follows: 41 (93.2%) drugstores dispensed antibiotics at demand level 1, 1 (2.3%) drugstore at demand level 2, and 2 (4.5%) drugstores at demand level 3. The most commonly dispensed antibiotics without a prescription were levofloxacin 24.4% (*n* = 11), ciprofloxacin 20% (*n* = 9), and azithromycin 17.8% (*n* = 8).

### 3.4 Dispensing of non-prescription antibiotics for diarrheal infection

SPs presented diarrheal cases at 44 drugstores, and antibiotics were dispensed by 43 of them. Antibiotics were dispensed at demand levels 1, 2, and 3 from 35 (81.4%), 7 (16.3%), and 1 (2.3%) drugstore, respectively. The most commonly dispensed antibiotics for diarrhea without prescription were metronidazole 40.9% (*n* = 18), the combinations of metronidazole and ciprofloxacin 27.3% (*n* = 12), and ciprofloxacin alone 25% (*n* = 11). There was no significant difference in antibiotic dispensing rate between flu and diarrhea (*p* = 0.987).

### 3.5 Dispensing of non-prescription antibiotics for sore throat

SPs presented a case of sore throat at 40 different drugstores. All of these offered antibiotics without a prescription. Among these, antibiotics were obtained without a prescription at demand levels 1 and 3 in 37 (81.4%) and 3 (7.5%) drugstores, respectively. The most commonly dispensed antibiotics included levofloxacin 32.5% (*n* = 13), erythromycin 20% (*n* = 8), and azithromycin 12.5% (*n* = 5). There was no significant difference in antibiotic dispensing rate between flu and sore throat (*p* = 0.998).

### 3.6 Dispensing of non-prescription antibiotics for UTIs

SPs presented the UTIs scenario at 44 drugstores. Antibiotics were obtained from 40 (90.9%) drugstores, with 47.5% dispensed without a prescription at demand level 1, 30% at level 2, and 22.5% at level 3. The most commonly dispensed antibiotic for UTI was ciprofloxacin 70.5% (*n* = 31). There was no significant difference in antibiotic dispensing between UTIs and flu (*p* = 0.193).

### 3.7 Association between drugstore category, location of drugstore, and simulated scenarios with the extent of non-prescription antibiotics dispensing

In four drugstores where a pharmacist was present at the time of visit, all of them dispensed antibiotics without a prescription. So, a bivariate analysis of pharmacist availability with the extent of antibiotic dispensing was not performed. *P*-values were determined by using Fisher's test in bivariate analysis between drugstore category, type of drugstore, location of drugstore, age of staff, with the extent of antibiotic dispensing. Location of the drug store was found to be statistically significant (*p* = 0.021). Bivariate analysis of characteristics of drug stores and drug store staff is given in Table 4. Due to the chance of confounding, the drugstore category and simulated scenarios were also included with the location of the drugstore in the multivariate analysis. The findings demonstrate that when pharmacy and medical stores were compared, there was 3.8 times more chance of antibiotics dispensing without prescription in medical stores than in pharmacies (95% CI: 0.392–38.834, AOR:3.899), but their association was not statistically significant (*p* = 0.246). Drugstores located in front of the hospitals are 10.1 times less likely to dispense antibiotics without a prescription than drugstores located in the community settings (95%CI:0.016–0.619, AOR:0.099). *P*-value for drugstore location in multivariate analysis was found to be 0.099, so drugstore location is significantly impacting antibiotic dispensing rate without a prescription. The findings demonstrate that when flu is compared with diarrhea, sore throat, and UTIs, there was no difference in the extent of antibiotic dispensing without prescription, and also none of the simulated scenarios show a statistically significant association with non-prescription antibiotic dispensing. It is estimated that in UTIs, drugstores are less likely to dispense antibiotics without a prescription when compared with flu (95%CI:0.016–1.823, AOR:0.143). Multivariate analysis of characteristics of drugstore and drugstore staff is given in Table 5.

**Table 4:** Bivariate analysis of characteristics of drugstores and drugstore staff with the extent of antibiotic dispensing without a prescription.

Variable	Total	Antibiotic dispensed without a prescription		Bivariate Analysis		
	(n = 173)	Yes (*n* = 167)	No (n = 6)	p-value	95% CI	COR
Drugstore category						
Pharmacy	93 (53.8%)	88 (94.6%)	5 (5.4%)			Reference
Medical store	80 (46.2%)	79 (98.8%)	1 (1.3%)	**0.219**⁎	0.025–1.948	0.223

Type of drugstore						
Individual	163 (94.2%	158 (96.9%)	5 (3.1%)			Reference
Chain	) 10 (5.8%)	9 (90%)	1 (10%)	**0.304**⁎	0.030–2.701	0.285

Location of drugstores						
Community	153 (88.4%	150 (98%)	3 (1.9%)			Reference
In front of the hospital	) 20 (11.6%)	17 (85%)	3 (1.5%)	**0.021**⁎⁎	0.021–0.606	0.113

Age of staff						
< 30 years	70 (40.5%)	67 (95.7%)	3 (4.3%)			Reference
≥ 30 years	103 (59.5%)	100 (97.1%)	3 (2.9%)	**0.687**⁎	0.096–7.617	1.493

Simulated scenarios						
Flu	45 (26%)	44 (97.8%)	1 (2.2%)			Reference
Diarrhea	44 (25.4%)	43 (97.7%)	1 (2.3%)	0.987	0.059–16.127	0.977
Sore throat	40 (23.1%)	40 (100%)	0	0.998	-	0.000
UTIs	44 (25.4%)	40 (90.9%)	4 (9.1%)	0.193	0.024–2.119	0.227

⁎ Fischer's p-value.

⁎⁎ Fischer's *p*-value and significant.

**Table 5:** Bivariate and Multivariate analysis with characteristics of drugstores and non-prescription antibiotic dispensing.

Bivariate Analysis				Multivariate Analysis		
Variables	95%CI	COR	*p*-value	95% CI	AOR	p-value
Pharmacy		Reference				
Medical store	0.025–1.948	0.223	0.175	0.392–38.834	3.899	0.246

Location						
Community		Reference				
In front of hospital	0.021–0.606	0.113	0.011	0.016–0.619	0.099	0.013

Simulated Scenarios						
Flu		Reference				
Diarrhea	0.059–16.127	0.977	0.987	0.049–16.308	0.896	0.941
Sore throat	–	–	0.998	–	–	0.998
UTIs	0.024–2.119	0.227	0.193	0.016–1.823	0.170	0.143

### 3.8 Medicines dispensed other than antibiotics

In case of flu, paracetamol, paracetamol with cetirizine, paracetamol with tramadol, and mefenamic acid were commonly dispensed medicines with antibiotics. Loperamide, loperamide with ORS, and loperamide with omeprazole were commonly dispensed for diarrhea. In case of sore throat, paracetamol, paracetamol with cetirizine, dextromethorphan with chlorpheniramine maleate, and paracetamol with mefenamic acid were commonly dispensed medicines, with antibiotics. In case of UTIs, cranberry juice, cranberry juice with sodium acid citrate, cranberry with diclofenac sodium, and cranberry with diclofenac potassium were commonly dispensed medicines with antibiotics.

## 4 Discussion

The study's findings provided valuable insights into how often and in what patterns non-prescription antibiotics are dispensed for common infections. Also, it highlights a significant connection between geographical location, different simulated scenarios, and the level of antibiotic dispensing.

According to antibiotic dispensing guidelines in the majority of LMICs, including Pakistan, antibiotics should be dispensed against a valid prescription issued by a registered medical practitioner.^21^ Despite the clarity of these prescription-only policies, the critical challenge is their implementation at the community level. The operational failure to enforce these regulations creates a significant gap between policies and practices, directly facilitates self-medication and irrational use that can foster resistance, contributes to the resources' wastage, treatment failure, and an increased risk of adverse drug reactions.^37^ Pakistan provides a critical example of the enforcement gap, where the dispensing of antibiotics without a prescription is widespread. This study found that dispensing of antibiotics without a prescription is prevalent (96.5%), underscoring a serious public health challenge. The findings are not an anomaly but confirm a pervasive, nationwide issue, as another multi-center cross-sectional study reported similar dispensing practices.^25^ This alarming rate of non-prescription antibiotics dispensing is higher than many nearby developing countries having almost similar economic status, including Bangladesh,^38^ India,^39^ and Vietnam.^40^ This depicts a very concerning health challenge, restricting the achievement of Sustainable Development Goal 3 (Health and Well-being). The national health sector is already fragile and unable to provide even basic health needs, lacking healthcare staff, necessary equipment, basic medicines, and valuable diagnostic testing services.^41^ So, in such situations, if the irrational antibiotic dispensing practices were not controlled, the resulting cases of AMR infections would be an additional burden on the healthcare system. Considering the evidence from developed countries that have effectively minimized unauthorized dispensing and associated irrational use by rigorously implementing the prescription-only regulations of antibiotic dispensing,^42^, ^43^Pakistan must enforce the strict implementation of these regulations to alleviate the burden of AMR on its healthcare system and safeguard public health.

The availability of a qualified person at the drugstores is a basic legal and ethical requirement,^21^ as they are the key players to limit non-prescription antibiotic dispensing. Unfortunately, it was found to be violated, as the qualified personnel were not available at most of the drugstores (94.8%). This may lead to the dispensing of antibiotics without professional oversight, directly compromising patient safety and the quality of pharmaceutical care. Limited availability of pharmacists may be attributed to several factors, including lenient law enforcement, low salaries for full-time pharmacists, and ineffective oversight by drug inspectors.^12^ Additionally, the practice of employing only one pharmacist to cover long operating hours, seven days a week, makes it nearly impossible for them to be present at all times. The provision of antibiotics without a prescription by the qualified personnel was very concerning. The involvement of the qualified personnel in such malpractices is a question mark on their professional responsibility. Pharmacists, as a last-line safeguard in the healthcare chain, possess a unique opportunity to avert antibiotics misuse by counseling patients on the difference between viral and bacterial infection, recommending symptomatic care, and ethically refusing to dispense when antibiotics are unwarranted.^44^ But the statistics of the present study indicated that pharmacy personnel are not prepared to redefine the narrative from high usage to actively practicing responsible antimicrobial stewardship. A potential reason for this is the involvement of non-professionals as owners of multiple pharmacy business licenses. These owners may pressure licensed professionals for economic gains, potentially compromising their professional decisions.^45^

Another concern revealed in the present study is that most of the antibiotics (79%) were dispensed on recommendations of drugstore staff (Demand level 1), without a customer's specific request for antibiotics, either because of their insufficient knowledge or financial considerations. This high rate of unassessed antibiotic dispensing carries severe clinical implications, as it bypasses the professional responsibility to evaluate a patient's need, thereby directly increasing the risk of inappropriate antibiotic use.^45^ Several simulated studies demonstrated that antibiotics were mostly sold to meet the customer demand rather than following the advice of attending staff.^46^, ^47^ It is similar to what was observed at demand level 3, where out of sixteen drugstores, six dispensed ciprofloxacin, suggesting that requesting antibiotics by using their names may influence the selection of antibiotics by drugstore staff. It shows that drugstore staff tend to comply with patients' antibiotic requests rather than assessing their clinical needs. In LMICs, including Pakistan, patients prefer to self-treat common infections because of their poor socio-economic status, high consultancy fees of clinicians, and unavailability of time and other resources.^19^, ^48^ The easy availability of antibiotics without a prescription at drugstores to retain and gain the loyalty of patients is a major contributing factor to irrational use. On the one side, where such malpractices pose serious health concerns, the terrifying angle of this picture is the provision of broad-spectrum antibiotics for self-limiting infections, which can easily be managed with home-based remedies. Ciprofloxacin, levofloxacin, and azithromycin have been placed in the Watch group of AWaRe classification of antibiotics provided by WHO,^49^ which means their use should be limited to specific indicated infections that can't be controlled by Access group antibiotics. The higher dispensing rate of these broad-spectrum antibiotics results in two types of health issues: one is the cross-resistance of both fluoroquinolones and macrolides, and the second is the emergence of Watch group-resistant infections,^11^ leaving behind only a few treatment options which may further complicate the conditions. The usage of fluoroquinolones in Pakistan has increased significantly over the years, prompting the Drug Registration Board to issue an advisory due to serious side effects, including tendon rupture, nerve damage, and peripheral neuropathy.^50^ Considering the serious side effects of fluoroquinolones, the UK's Medicines and Healthcare Products Regulatory Agency (MHRA) has issued strict limitations on their use for minor self-limiting and non-bacterial infections, due to associated long-term adverse effects.^51^ This study revealed that despite so many national and international restrictions, fluoroquinolones are still the most frequently dispensed antibiotics in Pakistan.

Interestingly, the location of drugstores appeared to play a key role in antibiotics dispensing for common infections. Drugstores located in front of hospitals were 10.1 times less likely to dispense antibiotics without a prescription compared to those in community areas (95% CI: 0.016–0.619, AOR = 0.099). This may be due to the competitive environment among community drugstores that influences them strongly to dispense antibiotics without a prescription to retain customers and prevent losing business to competitors. Besides this, drugstores located near hospitals work under frequent scrutiny from regulatory authorities, while community drugstore often lack such frequent oversight.^52^ Therefore, regulatory authorities must strengthen surveillance in community settings by establishing specialized teams to inspect and enforce compliance. Furthermore, equipping drugstore staff with better tools, improved communication skills, and clear guidelines will significantly enhance collective resilience against the looming threats of AMR.

### 4.1 Strengths and limitations

The strengths of this study lie in the use of the SP method, which minimizes bias and enhances the reliability of findings by ensuring anonymity. Accurate data on non-prescription antibiotic dispensing rates provides valuable insights for policymakers. However, this study has some limitations, including its focus on an urban setting in Bahawalpur, limiting generalizability to rural areas and the entire country. Although systematic random sampling was adopted to obtain a sample of 173, it is relatively a modest representation of the national pharmacy landscape. Furthermore, this study records data on specific case scenarios, and it may not be possible to extrapolate to other conditions. Lastly, the study did not assess variability in dispensing practices through repeated visits, nor did it explore the underlying reasons for non-prescription antibiotics sales. Despite these limitations, the study offers meaningful guidance for future research and policy development.

## 5 Conclusion

Despite regulations, sales of antibiotics without a prescription are alarming, which can accelerate resistance and jeopardize the availability of antibiotics in the country. In most cases, it is easy to obtain broad-spectrum antibiotics such as fluoroquinolones and macrolides for common infections, which erodes the efficacy of these most critical drugs, introduces long-term risks, compromises treatment protocols, and escalates the mortality rates. These current practices of dispensing non-prescription antibiotics highlight the urgency for policymakers to develop multifaceted approaches, including the establishment of stricter regulations, implementation of both prescription-only policy and qualified person on duty rules, staff training, and launching impactful public education initiatives.

## CRediT authorship contribution statement

**Arsha Khan:** Writing – review & editing, Writing – original draft, Visualization, Validation, Data curation. **Muhammad Awais Fareed:** Writing – review & editing, Writing – original draft, Data curation. **Daniyal Rauf:** Writing – review & editing, Writing – original draft, Data curation. **Muhammad Ameer Usama:** Writing – review & editing, Writing – original draft, Data curation. **Mubashra Rehman:** Writing – review & editing, Writing – original draft. **Neha Sheerin:** Writing – review & editing, Writing – original draft, Visualization. **Atif Usman:** Writing – review & editing, Writing – original draft, Supervision, Formal analysis. **Zainub Khan:** Writing – review & editing, Writing – original draft, Visualization, Data curation. **Imran Masood:** Writing – review & editing, Writing – original draft, Supervision, Formal analysis. **Jamshaid Akbar:** Writing – review & editing, Writing – original draft, Supervision, Formal analysis, Conceptualization. **Ali Ahmed:** Writing – review & editing, Writing – original draft, Supervision.

## Ethical approval

The study was approved by Pharmacy Human Ethics Committee (PHEC), Islamia University of Bahawalpur, Pakistan (Ref No. 244–2025-/PHEC, May 12, 2025).

## Funding statement

The project has not been funded by the institution or any other organization.

## Declaration of competing interest

The authors declare that they have no known competing financial interests or personal relationships that could have appeared to influence the work reported in this paper.

## Data Availability

Data can be made available upon request to the corresponding author.

## References

[1] Deori C., Sonowal T., Das M. (2024). Antimicrobial resistance: a looming threat to public health and global well-being. Ind J Commun Fam Med.

[2] Aljeldah M.M. (2022). Antimicrobial resistance and its spread is a global threat. Antibiotics.

[3] Khan M.S., Iqbal M.S., Sonehri H. (2025). Antibiotic resistance: a looming threat to public health. Res J Psychol.

[4] Rimal P., Sah A.K., Alam K. (2025). Dispensing practices of antibiotics among community pharmacies of Bharatpur, Nepal: a simulated patient cross-sectional study. J Young Pharm.

[5] Jonas O.B., Irwin A., Berthe F.C.J. (2023). https://documents1.worldbank.org/curated/en/323311493396993758/pdf/final-report.

[6] Torres N.F., Solomon V.P., Middleton L.E. (2020). Pharmacists practices for non-prescribed antibiotic dispensing in Mozambique. Pharmacy Practice (Granada).

[7] Belachew S.A., Hall L., Selvey L.A. (2022). Community drug retail outlet staffs knowledge, attitudes and practices towards non-prescription antibiotics use and antibiotic resistance in the Amhara region, Ethiopia with a focus on non-urban towns. Antimicrob Resist Infect Control.

[8] World Health Organization (2015). https://iris.who.int/handle/10665/194460.

[9] Llor C., Benkő R., Bjerrum L. (2024). Global restriction of the over-the-counter sale of antimicrobials: does it make sense?. Front Public Health.

[10] Sambakusi C.S. (2019). Knowledge, attitudes and practices related to self-medication with antimicrobials in Lilongwe, Malawi. Malawi Med J.

[11] Auta A., Hadi M.A., Oga E. (2019). Global access to antibiotics without prescription in community pharmacies: a systematic review and meta-analysis. J Infect.

[12] Amin M., Chewning B. (2017). Pharmacies without pharmacists: absenteeism plagues pharmacies in developing countries. Res Social Adm Pharm.

[13] Miller R., Goodman C. (2016). Performance of retail pharmacies in low-and middle-income Asian settings: a systematic review. Health Policy Plan.

[14] Majid Aziz M., Haider F., Rasool M.F. (2021). Dispensing of non-prescribed antibiotics from community pharmacies of Pakistan: a cross-sectional survey of pharmacy staffs opinion. Antibiotics.

[15] Torres N.F., Chibi B., Kuupiel D. (2021). The use of non-prescribed antibiotics; prevalence estimates in low-and-middle-income countries. A systematic review and meta-analysis. Arch Public Health.

[16] Li J., Zhou P., Wang J. (2023). Worldwide dispensing of non-prescription antibiotics in community pharmacies and associated factors: a mixed-methods systematic review. Lancet Infect Dis.

[17] Klein E.Y., Van Boeckel T.P., Martinez E.M. (2018). Global increase and geographic convergence in antibiotic consumption between 2000 and 2015. Proc Natl Acad Sci.

[18] Jang (2016). Rise in use of antibiotics in pakistan. https://jang.com.pk/news/192118-use-of-antibiotics-rise-in-pakistan.

[19] Haseeb A., Bilal M. (2016). Prevalence of using non prescribed medications in economically deprived rural population of Pakistan. Arch Public Health.

[20] Imtiaz F., Hafeez A., Ashraf F. (2017). Antibiotic dispensing & prescription pattern in pharmacies of Islamabad & Rawalpindi: Pakistan. Int J Collab Res Intern Med Public Health.

[21] Drug Regulatory Authority of Pakistan (2026). The Drugs Act 1976. https://www.dra.gov.pk/about-us/legislation/acts/.

[22] Ministry of National Health Services Regulations & Coordination Government of Pakistan (2017). Antimicrobial resistance national action plan Pakistan. https://www.nih.org.pk/wp-content/uploads/2018/08/AMR-National-Action-Plan-Pakistan.pdf.

[23] Saleem Z., Hassali M.A., Hashmi F.K. (2018). Pakistan’s national action plan for antimicrobial resistance: translating ideas into reality. Lancet Infect Dis.

[24] Ahmad T., Khan F.U., Ali S. (2022). Assessment of without prescription antibiotic dispensing at community pharmacies in Hazara division, Pakistan: a simulated clients study. PloS One.

[25] Saleem Z., Hassali M.A., Godman B. (2020). Sale of WHO AWaRe groups antibiotics without a prescription in Pakistan: a simulated client study. J Pharm Policy Pract.

[26] Hashmi F.K., Khadka S., Khan M.h.M. (2021).

[27] Björnsdottir I., Granas A.G., Bradley A. (2020). A systematic review of the use of simulated patient methodology in pharmacy practice research from 2006 to 2016. Int J Pharm Pract.

[28] Sedgwick P. (2012). The Hawthorne effect. BMJ.

[29] Malik U.R., Chang J., Hashmi F. (2021). A simulated client exploration of nonprescription dispensing of antibiotics at drugstores for pediatric acute diarrhea and upper respiratory infection in Lahore, Pakistan. Infect Drug Resist.

[30] Hasan R., Mohey-ud-Din G. (2019). Social development in Punjab–Pakistan: a district level analysis. J Asian Dev Stud.

[31] Elkhadry S.W., Tahoon M.A.H. (2024). Health literacy and its association with antibiotic use and knowledge of antibiotic among Egyptian population: cross sectional study. BMC Public Health.

[32] Park J.S., Page A., Clifford R. (2024). Refining the CRiSPHe (checklist for reporting research using a simulated patient methodology in health): a Delphi study. Int J Pharm Pract.

[33] Mohsin M. (2015). Rapid urban growth and change in urban and municipal limits of Bahawalpur city, Pakistan: a spatio-periodical discourse. J Basic Appl Sci.

[34] Centralized Drug Sale Licensing – Public Portal (CDSL-PP) Valid POS. https://eservices-cdsl.pshealthpunjab.gov.pk/auth/login.

[35] Raosoft (2004). Sample Size Calculator. http://www.raosoft.com/samplesize.html.

[36] Holloway K.A., Kotwani A., Batmanabane G. (2017). Antibiotic use in South East Asia and policies to promote appropriate use: reports from country situational analyses. BMJ.

[37] Alhomoud F., Almahasnah R., Alhomoud F.K. (2018). “You could lose when you misuse”–factors affecting over-the-counter sale of antibiotics in community pharmacies in Saudi Arabia: a qualitative study. BMC Health Serv Res.

[38] Islam M.A., Akhtar Z., Hassan M.Z. (2022). Pattern of antibiotic dispensing at pharmacies according to the WHO access, watch, reserve (AWaRe) classification in Bangladesh. Antibiotics Basel.

[39] Nafade V., Huddart S., Sulis G. (2019). Over-the-counter antibiotic dispensing by pharmacies: a standardised patient study in Udupi district, India. BMJ Glob Health.

[40] Do T.A., Quan P.B., Le T.T.-B. (2025). Antibiotic dispensing without a prescription across community pharmacies: a simulated patient study. Explor Res Clin Social Pharm.

[41] Muhammad Q., Eiman H., Fazal F. (2023). Healthcare in Pakistan: navigating challenges and building a brighter future. Cureus.

[42] Zapata-Cachafeiro M., Piñeiro-Lamas M., Guinovart M.C. (2018). Magnitude and determinants of antibiotic dispensing without prescription in Spain: a simulated patient study. J Antimicrob Chemother.

[43] Ashiru-Oredope D., Cunningham N., Casale E. (2023). Reporting englands progress towards the ambitions in the UK action plan for antimicrobial resistance: the English surveillance programme for antimicrobial utilisation and resistance (ESPAUR). J Antimicrob Chemother.

[44] Zerbinato F., Cunningham S., Tonna A. (2025). Antimicrobial stewardship interventions involving community pharmacy teams: a scoping. Pharm Educ.

[45] Asghar S., Atif M., Mushtaq I. (2020). Factors associated with inappropriate dispensing of antibiotics among non-pharmacist pharmacy workers. Res Social Adm Pharm.

[46] Chang J., Ye D., Lv B. (2017). Sale of antibiotics without a prescription at community pharmacies in urban China: a multicentre cross-sectional survey. J Antimicrob Chemother.

[47] Chang J., Xu S., Zhu S. (2019). Assessment of non-prescription antibiotic dispensing at community pharmacies in China with simulated clients: a mixed cross-sectional and longitudinal study. Lancet Infect Dis.

[48] Zeid W., Hamed M., Mansour N. (2020). Prevalence and associated risk factors of self-medication among patients attending El-Mahsama family practice center, Ismailia, Egypt. Bull Natl Res Cent.

[49] World Health Organization (2026). WHO antibiotic categorization. https://aware.essentialmeds.org/groups.

[50] Global Antibiotic Resistance Partnership G.A.R (2026). Situation analysis report on antimicrobial resistance Pakistan. https://onehealthtrust.org/wp-content/uploads/2018/03/Situational-Analysis-Report-on-Antimicrobial-Resistance-in-Pakistan.pdf.

[51] Chris Dall M. (2024). UK tightens restrictions on fluoroquinolone use. https://www.cidrap.umn.edu/antimicrobial-stewardship/uk-tightens-restrictions-fluoroquinolone-use.

[52] Hussain A., Ibrahim M.I., Baber Z.-u.-D. (2012). Compliance with legal requirements at community pharmacies: a cross sectional study from Pakistan. Int J Pharm Pract.

