# Supplementary material for: Non-prescription antibiotics dispensing for common infections using simulated patients
Source: Explor Res Clin Soc Pharm. 2026 Apr 24;23:100791. doi: 10.1016/j.rcsop.2026.100791 (PMC13185847; doi:10.1016/j.rcsop.2026.100791)
Supplement: Supplementary file 1 — Supplementary material [file mmc1.docx]

**Simulated Scenario 1 (Influenza) flu**

**Demand level 1:**

Simulated client will tell the pharmacy staff that he/she has a 25-year-old brother/sister having the following symptoms:

- Sudden onset
- May or mayn’t have fever
- Headache
- Chills
- Body aches
- Sneezing
- Coughing
- Sore throat
- Weakness

If an antibiotic is dispensed, then skip the other demand levels and memorize the antibiotic dispensed, and leave the pharmacy. Fill the questionnaire within 10 minutes after leaving the pharmacy.

If an antibiotic is not dispensed, then go to demand level 2.

**Demand level 2:**

Tell the pharmacy staff that you have had symptoms for 3 days. Ask him/her to dispense you some sort of antibiotic.

**Answers to the questions, if asked by pharmacy staff:**

**Taking any medicine**: No medicine, only using the steam of water.

**Any drug allergy**: No

**Medical history**: No

**Visited doctor or not**: No

**Why haven’t you visited the doctor?**

I can’t afford the doctor’s fee, or I think there is no need to see a doctor. It is just a minor illness. You give me some sort of antibiotic, I will do better; otherwise, I will see a doctor.

**Have prescription**: No

If an antibiotic is dispensed, then skip other demands and memorize the antibiotic dispensed, and leave the pharmacy. Fill the questionnaire within 10 minutes after leaving the pharmacy.

If an antibiotic is not dispensed, then go to demand level 3.

**Demand level 3:**

Give me azithromycin or co-amoxiclav, or ciprofloxacin.

If an antibiotic is dispensed, then memorize the antibiotic dispensed and leave the pharmacy. Fill the questionnaire within 10 minutes after leaving the pharmacy.

If an antibiotic is not dispensed, then ask about the reason for refusal and fill out the questionnaire within 10 minutes after leaving the pharmacy.

**Simulated Scenario 2 (Acute diarrhea)**

**Demand level 1:**

Simulated client will tell the pharmacy staff that he/she has a 25-year-old brother/sister having the following symptoms:

- Abdominal pain
- Nausea or vomiting or both
- Loose watery stool
- Fever

If an antibiotic is dispensed, then skip other demands and memorize the antibiotic dispensed, and leave the pharmacy. Fill the questionnaire within 10 minutes after leaving the pharmacy.

If antibiotic is not dispensed than go to demand level 2.

**Demand level 2:**

Tell the pharmacy staff that he/she has had symptoms for 3 days. Ask him/her to dispense you some sort of antibiotic.

**Answers to the questions if asked by pharmacy staff:**

**Taking any medicine**: No medicine, only using the steam of water.

**Any drug allergy**: No

**Medical history**: No

**Visited doctor or not**: No

**Why haven’t you visited the doctor?** I can’t afford the doctor’s fee.

I think there is no need to see the doctor. It is just a minor illness. You give me some sort of antibiotic, it will do better; otherwise, I will see a doctor.

**Have prescription**: No

If an antibiotic is dispensed, then skip other demands and memorize the antibiotic dispensed, and leave the pharmacy. Fill the questionnaire within 10 minutes after leaving the pharmacy.

If an antibiotic is not dispensed, then go to demand level 3.

**Demand level 3:**

Give me ciprofloxacin or cefixime or metronidazole.

If an antibiotic is dispensed, then memorize the antibiotic dispensed and leave the pharmacy. Fill the questionnaire within 10 minutes after leaving the pharmacy.

If an antibiotic is not dispensed, then ask about the reason for refusal and fill out the questionnaire within 10 minutes after leaving the pharmacy.

**Simulated Scenario 3 (Sore throat)**

**Demand level 1:**

Simulated client will tell the pharmacy staff that he/she has a 25-year-old brother/sister having the following symptoms:

- Painful and difficult swallowing
- Slight fever
- Dry and scratchy throat
- Cough
- Runny nose
- Hoarseness

If an antibiotic is dispensed, then skip other demands and memorize the antibiotic dispensed, and leave the pharmacy. Fill the questionnaire within 10 minutes after leaving the pharmacy.

If an antibiotic is not dispensed, then go to demand level 2.

**Demand level 2:**

Tell the pharmacy staff that he/she has had symptoms for 3 days. Ask him/her to dispense you some sort of antibiotic.

**Answers to the questions, if asked by pharmacy staff:**

**Taking any medicine**: No medicine, only using the steam of water.

**Any drug allergy**: No

**Medical history**: No

**Visited doctor or not**: No

**Why haven’t you visited the doctor?** I can’t afford the doctor’s fee.

I think there is no need to see a doctor. It is just a minor illness. You give me some sort of antibiotic, I will do better; otherwise, I will see a doctor.

**Have prescription**: No

If an antibiotic is dispensed, then skip other demands and memorize the antibiotic dispensed, and leave the pharmacy. Fill the questionnaire within 10 minutes after leaving the pharmacy.

If an antibiotic is not dispensed, then go to demand level 3.

**Demand level 3:**

Give me azithromycin or co-amoxiclav, or ciprofloxacin.

If an antibiotic is dispensed, then memorize the antibiotic dispensed and leave the pharmacy. Fill the questionnaire within 10 minutes after leaving the pharmacy.

If an antibiotic is not dispensed, then ask about the reason for refusal and fill out the questionnaire within 10 minutes after leaving the pharmacy.

**Simulated Scenario 4 (Urinary tract infections)**

**Demand level 1:**

Simulated client will tell the pharmacy staff that he/she has a 25-year-old sister having the following symptoms:

- Flank pain
- Fever
- Frequent urination for two days
- cloudy, [bloody](https://www.niddk.nih.gov/health-information/urologic-diseases/hematuria-blood-urine), or strong-smelling urine

If an antibiotic is dispensed, then skip other demands and memorize the antibiotic dispensed, and leave the pharmacy. Fill the questionnaire within 10 minutes after leaving the pharmacy.

If an antibiotic is not dispensed, then go to demand level 2.

**Demand level 2:**

Tell the pharmacy staff that she has had symptoms for 2 days. Ask him/her to dispense you some sort of antibiotic.

**Answers to the questions, if asked by pharmacy staff:**

**Taking any medicine**: No medicine, only using the steam of water.

**Any drug allergy**: No

**Medical history**: No

**Visited doctor or not**: No

**Why haven’t you visited the doctor?** I can’t afford the doctor’s fee.

I think there is no need to see the doctor. It is just a minor illness. You give me some sort of antibiotic, I will do better; otherwise, I will see a doctor.

**Have prescription**: No

If an antibiotic is dispensed, then skip other demands and memorize the antibiotic dispensed, and leave the pharmacy. Fill the questionnaire within 10 minutes after leaving the pharmacy.

If an antibiotic is not dispensed, then go to demand level 3.

**Demand level 3:**

Give me ciprofloxacin or levofloxacin or cefixime.

If an antibiotic is dispensed, then memorize the antibiotic dispensed and leave the pharmacy. Fill the questionnaire within 10 minutes after leaving the pharmacy.

If an antibiotic is not dispensed, then ask about the reason for refusal and fill out the questionnaire within 10 minutes after leaving the pharmacy.
